# *N*-Benzyl-3,5-dide­oxy-3,5-imino-1,2-*O*-isopropyl­idene-β-l-lyxofuran­ose

**DOI:** 10.1107/S1600536812030656

**Published:** 2012-07-10

**Authors:** David S. Edgeley, Sarah F. Jenkinson, Gabriel Lenagh-Snow, Catherine Rutherford, George W. J. Fleet, Amber L. Thompson

**Affiliations:** a Department of Chemical Crystallography, Chemistry Research Laboratory, University of Oxford, Oxford OX1 3TA, England; b Department of Organic Chemistry, Chemistry Research Laboratory, University of Oxford, Oxford OX1 3TA, England

## Abstract

X-ray crystallography confirmed the formation, structure and relative stereochemistry of the title compound, C_15_H_19_NO_3_, which contains a sterically congested four-membered azetidine ring system. The absolute configuration was determined by the use of l-arabinose as the starting material.

## Related literature

For related literature on azetidines, see: Krämer *et al.* (1997 ▶); Michaud *et al.* (1997*a* ▶,*b* ▶); Dekaris & Reissig (2010 ▶); Soengas *et al.* (2011 ▶); Jenkinson *et al.* (2011 ▶); Lenagh-Snow *et al.* (2011 ▶, 2012 ▶); Lee *et al.* (2012 ▶). For related literature on imino­sugars, see: Asano *et al.* (2000 ▶); Watson *et al.* (2001 ▶). For details of the cryostat, see: Cosier & Glazer (1986 ▶). For details of hydrogen refinement, see: Cooper *et al.* (2010 ▶). For references to the Chebychev polynomial, see: Prince (1982 ▶); Watkin (1994 ▶).
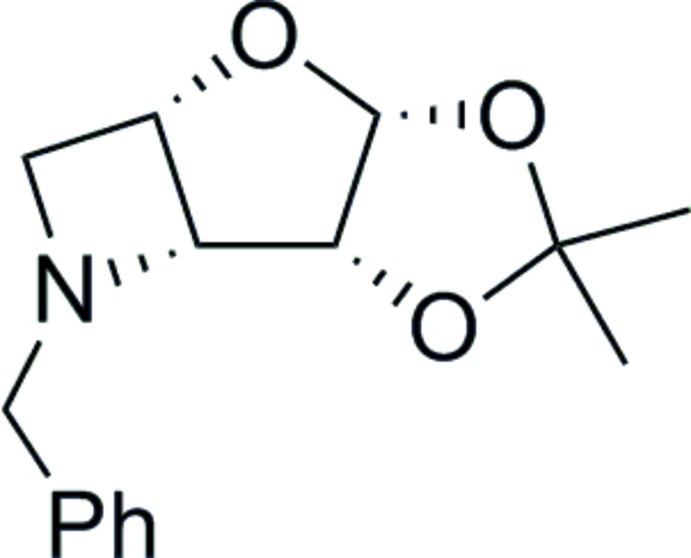


## Experimental

### 

#### Crystal data

C_15_H_19_NO_3_
*M* *_r_* = 261.32
Monoclinic, 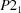

*a* = 9.1674 (2) Å
*b* = 5.7551 (1) Å
*c* = 13.1112 (3) Å
β = 106.9544 (8)°
*V* = 661.67 (2) Å^3^
*Z* = 2
Mo *K*α radiation
μ = 0.09 mm^−1^
*T* = 150 K
0.24 × 0.23 × 0.07 mm

#### Data collection

Nonius KappaCCD diffractometer
Absorption correction: multi-scan (*DENZO* and *SCALEPACK*; Otwinowski & Minor, 1997 ▶) *T*_min_ = 0.94, *T*_max_ = 0.99
13110 measured reflections
1638 independent reflections
1544 reflections with *I* > 2σ(*I*)
*R*_int_ = 0.014

#### Refinement

*R*[*F* ^2^ > 2σ(*F* ^2^)] = 0.033
*wR*(*F* ^2^) = 0.087
*S* = 0.94
1638 reflections
172 parameters
1 restraint
H-atom parameters constrained
Δρ_max_ = 0.17 e Å^−3^
Δρ_min_ = −0.18 e Å^−3^

### 

Data collection: *COLLECT* (Nonius, 2001 ▶); cell refinement: *DENZO* and *SCALEPACK* (Otwinowski & Minor, 1997 ▶); data reduction: *DENZO* and *SCALEPACK*; program(s) used to solve structure: *SIR92* (Altomare *et al.*, 1994 ▶); program(s) used to refine structure: *CRYSTALS* (Betteridge *et al.*, 2003 ▶); molecular graphics: *CAMERON* (Watkin *et al.*, 1996 ▶); software used to prepare material for publication: *CRYSTALS*.

## Supplementary Material

Crystal structure: contains datablock(s) global, I. DOI: 10.1107/S1600536812030656/lh5500sup1.cif

Structure factors: contains datablock(s) I. DOI: 10.1107/S1600536812030656/lh5500Isup2.hkl

Additional supplementary materials:  crystallographic information; 3D view; checkCIF report

## Figures and Tables

**Fig. 1.**
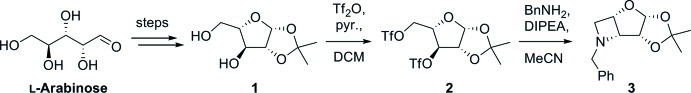
Synthetic Scheme.

**Fig. 2.**
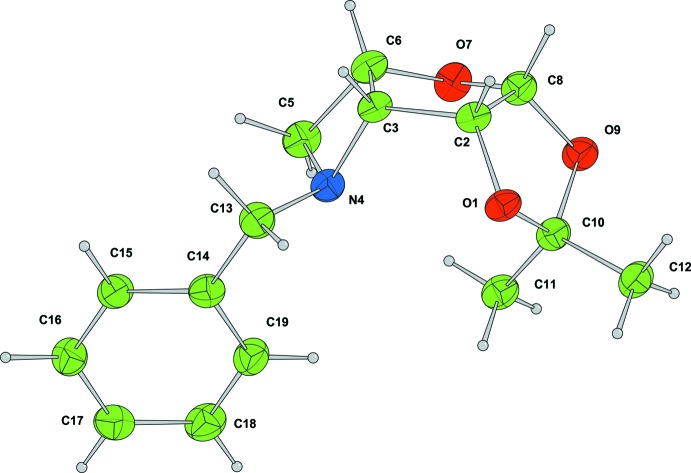
The title compound with displacement ellipsoids drawn at the 50% probability level. H atoms are shown as spheres of arbitrary radius.

**Fig. 3.**
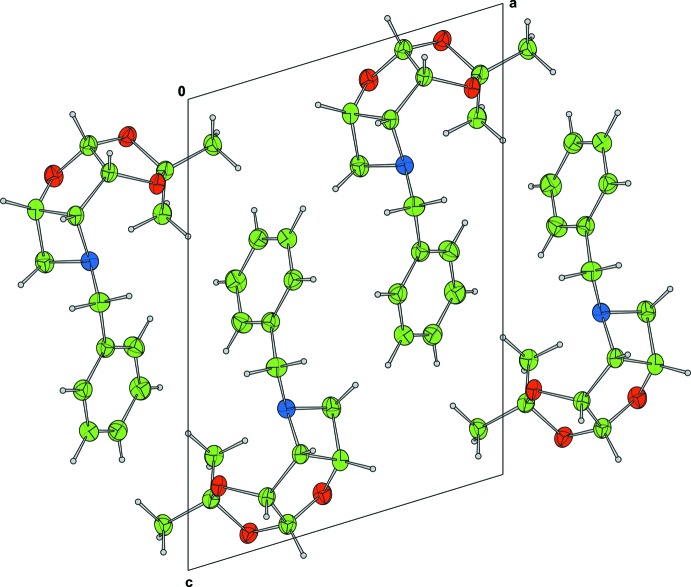
Packing diagram of the title compound projected along the b-axis.

## supplementary crystallographic information

### Comment

Azetidines (Michaud *et al.*, 1997*a*; Michaud *et al.*, 1997*b*; Dekaris & Reissig, 2010; Soengas *et al.*, 2011) are a relatively unstudied class of iminosugars (Asano *et al.*, 2000; Watson *et al.*, 2001, Michaud *et al.*, 1997*a*; Michaud *et al.*, 1997*b*; Dekaris & Reissig, 2010; Soengas *et al.*, 2011) but initial results (Krämer *et al.*, 1997; Lee *et al.*, 2012) have shown some interesting biological activity.

Azetidine formation can be achieved by the double displacement of ditriflates with amines (Jenkinson *et al.*, 2011; Lenagh-Snow *et al.*, 2011; Lenagh-Snow *et al.*, 2012). X-Ray crystallography confirmed the structure and relative stereochemistry of the formation of the title compound 3 (Fig. 1) from the displacement of a 3,5-*O*-ditriflate 2 with benzylamine. The absolute stereochemistry was determined by the use of L-arabinose as the starting material.

The five membered rings adopt envelope conformations with O7 and C10 out of the plane, and the azetidine ring adopts a puckered conformation (Fig. 2, Fig. 3).

### Experimental

The title compound was recrystallized from cyclohexane/pentane. [α]_D_^25^ +76.0 (*c* 0.50 in CHCl_3_); m.p. 337–339 K.

### Refinement

In the absence of significant anomalous scattering, Friedel pairs were merged and the absolute configuration was assigned from the starting material.

The H atoms were all located in a difference map, but those attached to carbon atoms were repositioned geometrically. The H atoms were initially refined with soft restraints on the bond lengths and angles to regularize their geometry (C—H in the range 0.93–0.98 Å) and *U*_iso_(H) (in the range 1.2–1.5 times *U*_eq_ of the parent atom), after which the positions were refined with riding constraints (Cooper *et al.*, 2010).

### Crystal data

**Table:**

C_15_H_19_NO_3_	*F*(000) = 280
*M**_r_* = 261.32	*D*_x_ = 1.312 Mg m^−^^3^
Monoclinic, *P*2_1_	Mo *K*α radiation, λ = 0.71073 Å
Hall symbol: P 2yb	Cell parameters from 1627 reflections
*a* = 9.1674 (2) Å	θ = 5–27°
*b* = 5.7551 (1) Å	µ = 0.09 mm^−^^1^
*c* = 13.1112 (3) Å	*T* = 150 K
β = 106.9544 (8)°	Plate, colourless
*V* = 661.67 (2) Å^3^	0.24 × 0.23 × 0.07 mm
*Z* = 2	

### Data collection

**Table:**

Nonius KappaCCD diffractometer	1544 reflections with *I* > 2σ(*I*)
Graphite monochromator	*R*_int_ = 0.014
ω scans	θ_max_ = 27.4°, θ_min_ = 5.4°
Absorption correction: multi-scan (*DENZO* and *SCALEPACK*; Otwinowski & Minor, 1997)	*h* = −11→11
*T*_min_ = 0.94, *T*_max_ = 0.99	*k* = −7→6
13110 measured reflections	*l* = −16→16
1638 independent reflections	

### Refinement

**Table:**

Refinement on *F*^2^	Primary atom site location: structure-invariant direct methods
Least-squares matrix: full	Hydrogen site location: difference Fourier map
*R*[*F*^2^ > 2σ(*F*^2^)] = 0.033	H-atom parameters constrained
*wR*(*F*^2^) = 0.087	Method, part 1, Chebychev polynomial, (Watkin, 1994, *P*rince, 1982) [*w*eight] = 1.0/[A_0_*T_0_(x) + A_1_*T_1_(x) ··· + A_n-1_]*T_n-1_(x)] where A_i_ are the Chebychev coefficients listed belo*w* and x = *F* /*F*max Method = Robust Weighting (*P*rince, 1982) W = [*w*eight] * [1-(delta*F*/6*sigma*F*)^2^]^2^ A_i_ are: 22.1 34.0 17.6 5.07
*S* = 0.94	(Δ/σ)_max_ = 0.0003286
1638 reflections	Δρ_max_ = 0.17 e Å^−^^3^
172 parameters	Δρ_min_ = −0.18 e Å^−^^3^
1 restraint	

### Special details

**Table:**

Experimental. The crystal was placed in the cold stream of an Oxford Cryosystems open-flow nitrogen cryostat (Cosier & Glazer, 1986) with a nominal stability of 0.1 K.

### Fractional atomic coordinates and isotropic or equivalent isotropic displacement parameters (Å^2^)

**Table:**

	*x*	*y*	*z*	*U*_iso_*/*U*_eq_	
O1	0.09793 (13)	0.5625 (2)	0.84062 (10)	0.0269	
C2	0.25050 (17)	0.6293 (3)	0.89568 (13)	0.0260	
C3	0.35745 (18)	0.6618 (3)	0.82587 (13)	0.0262	
N4	0.31206 (15)	0.5481 (3)	0.72006 (11)	0.0264	
C5	0.45568 (19)	0.4116 (4)	0.74729 (15)	0.0318	
C6	0.48402 (19)	0.4783 (3)	0.86494 (14)	0.0292	
O7	0.42651 (14)	0.3113 (3)	0.92411 (10)	0.0319	
C8	0.31601 (19)	0.4200 (3)	0.96665 (14)	0.0284	
O9	0.19278 (13)	0.2728 (3)	0.96037 (10)	0.0313	
C10	0.07349 (18)	0.3246 (3)	0.86343 (14)	0.0274	
C11	0.0820 (2)	0.1650 (3)	0.77364 (14)	0.0306	
C12	−0.07829 (19)	0.3058 (4)	0.88768 (15)	0.0333	
C13	0.2813 (2)	0.6967 (3)	0.62624 (14)	0.0309	
C14	0.2617 (2)	0.5526 (4)	0.52659 (14)	0.0288	
C15	0.3343 (2)	0.6152 (4)	0.45124 (14)	0.0320	
C16	0.3150 (2)	0.4828 (4)	0.35950 (14)	0.0353	
C17	0.2236 (2)	0.2866 (4)	0.34202 (15)	0.0346	
C18	0.1518 (2)	0.2208 (4)	0.41731 (16)	0.0379	
C19	0.1714 (2)	0.3523 (4)	0.50899 (15)	0.0343	
H21	0.2489	0.7722	0.9365	0.0300*	
H31	0.3962	0.8232	0.8266	0.0305*	
H51	0.4416	0.2480	0.7305	0.0388*	
H52	0.5313	0.4779	0.7148	0.0374*	
H61	0.5871	0.5325	0.9042	0.0348*	
H81	0.3666	0.4639	1.0430	0.0337*	
H112	0.0678	0.0054	0.7931	0.0457*	
H113	0.0002	0.2059	0.7092	0.0453*	
H111	0.1807	0.1782	0.7612	0.0454*	
H121	−0.0909	0.1489	0.9112	0.0506*	
H123	−0.1589	0.3444	0.8220	0.0494*	
H122	−0.0782	0.4156	0.9454	0.0507*	
H132	0.1871	0.7872	0.6208	0.0373*	
H131	0.3679	0.8067	0.6310	0.0372*	
H151	0.3977	0.7507	0.4630	0.0386*	
H161	0.3646	0.5290	0.3064	0.0422*	
H171	0.2100	0.2000	0.2782	0.0423*	
H181	0.0896	0.0854	0.4067	0.0458*	
H191	0.1208	0.3077	0.5589	0.0420*	

### Atomic displacement parameters (Å^2^)

**Table:**

	*U*^11^	*U*^22^	*U*^33^	*U*^12^	*U*^13^	*U*^23^
O1	0.0214 (5)	0.0238 (6)	0.0331 (6)	0.0007 (5)	0.0041 (4)	0.0028 (5)
C2	0.0234 (7)	0.0239 (8)	0.0291 (7)	−0.0006 (6)	0.0051 (6)	−0.0014 (7)
C3	0.0234 (7)	0.0242 (8)	0.0299 (7)	−0.0018 (6)	0.0063 (6)	−0.0014 (6)
N4	0.0255 (6)	0.0246 (7)	0.0293 (7)	−0.0002 (6)	0.0081 (5)	−0.0014 (6)
C5	0.0287 (8)	0.0290 (9)	0.0385 (9)	0.0023 (7)	0.0108 (7)	−0.0040 (8)
C6	0.0225 (7)	0.0289 (9)	0.0349 (8)	−0.0002 (7)	0.0063 (6)	−0.0003 (7)
O7	0.0293 (6)	0.0270 (6)	0.0393 (6)	0.0054 (5)	0.0100 (5)	0.0060 (6)
C8	0.0251 (7)	0.0274 (9)	0.0313 (8)	0.0014 (7)	0.0058 (6)	0.0036 (7)
O9	0.0264 (6)	0.0333 (7)	0.0311 (6)	−0.0017 (5)	0.0034 (5)	0.0078 (5)
C10	0.0248 (7)	0.0257 (8)	0.0299 (8)	−0.0007 (7)	0.0054 (6)	0.0049 (7)
C11	0.0291 (8)	0.0254 (8)	0.0361 (8)	−0.0019 (7)	0.0077 (7)	0.0013 (7)
C12	0.0264 (7)	0.0366 (10)	0.0376 (8)	−0.0030 (8)	0.0105 (6)	0.0037 (8)
C13	0.0365 (8)	0.0249 (8)	0.0318 (8)	−0.0035 (8)	0.0106 (7)	−0.0007 (7)
C14	0.0268 (7)	0.0279 (9)	0.0307 (8)	0.0010 (7)	0.0066 (6)	−0.0015 (7)
C15	0.0289 (8)	0.0330 (10)	0.0326 (8)	−0.0028 (8)	0.0069 (6)	0.0022 (7)
C16	0.0307 (8)	0.0430 (12)	0.0337 (9)	0.0009 (8)	0.0116 (7)	0.0003 (8)
C17	0.0306 (8)	0.0374 (10)	0.0342 (8)	0.0042 (8)	0.0070 (7)	−0.0071 (8)
C18	0.0374 (9)	0.0322 (10)	0.0447 (10)	−0.0062 (8)	0.0129 (8)	−0.0080 (9)
C19	0.0357 (9)	0.0317 (10)	0.0379 (9)	−0.0066 (8)	0.0147 (7)	−0.0034 (8)

### Geometric parameters (Å, º)

**Table:**

O1—C2	1.4271 (19)	C11—H112	0.972
O1—C10	1.433 (2)	C11—H113	0.981
C2—C3	1.535 (2)	C11—H111	0.967
C2—C8	1.534 (2)	C12—H121	0.972
C2—H21	0.984	C12—H123	0.983
C3—N4	1.480 (2)	C12—H122	0.985
C3—C6	1.542 (2)	C13—C14	1.513 (2)
C3—H31	0.994	C13—H132	0.993
N4—C5	1.484 (2)	C13—H131	1.003
N4—C13	1.457 (2)	C14—C15	1.390 (2)
C5—C6	1.536 (2)	C14—C19	1.399 (3)
C5—H51	0.967	C15—C16	1.391 (3)
C5—H52	0.990	C15—H151	0.957
C6—O7	1.429 (2)	C16—C17	1.385 (3)
C6—H61	0.986	C16—H161	0.974
O7—C8	1.435 (2)	C17—C18	1.389 (3)
C8—O9	1.395 (2)	C17—H171	0.950
C8—H81	1.005	C18—C19	1.387 (3)
O9—C10	1.4456 (19)	C18—H181	0.952
C10—C11	1.513 (3)	C19—H191	0.941
C10—C12	1.519 (2)		
			
C2—O1—C10	109.99 (13)	O9—C10—C12	107.79 (14)
O1—C2—C3	115.67 (13)	O1—C10—C12	108.68 (15)
O1—C2—C8	104.34 (14)	C11—C10—C12	112.21 (15)
C3—C2—C8	104.59 (14)	C10—C11—H112	109.1
O1—C2—H21	109.4	C10—C11—H113	108.9
C3—C2—H21	109.7	H112—C11—H113	109.0
C8—C2—H21	113.1	C10—C11—H111	110.3
C2—C3—N4	116.83 (13)	H112—C11—H111	108.8
C2—C3—C6	105.56 (14)	H113—C11—H111	110.7
N4—C3—C6	89.27 (13)	C10—C12—H121	109.5
C2—C3—H31	113.4	C10—C12—H123	107.6
N4—C3—H31	115.1	H121—C12—H123	111.0
C6—C3—H31	113.9	C10—C12—H122	108.6
C3—N4—C5	91.22 (12)	H121—C12—H122	109.0
C3—N4—C13	117.65 (15)	H123—C12—H122	111.1
C5—N4—C13	116.97 (14)	N4—C13—C14	110.62 (16)
N4—C5—C6	89.30 (13)	N4—C13—H132	108.6
N4—C5—H51	114.2	C14—C13—H132	110.1
C6—C5—H51	116.3	N4—C13—H131	111.3
N4—C5—H52	112.1	C14—C13—H131	107.0
C6—C5—H52	113.7	H132—C13—H131	109.2
H51—C5—H52	109.9	C13—C14—C15	120.71 (17)
C3—C6—C5	86.95 (13)	C13—C14—C19	120.65 (16)
C3—C6—O7	106.25 (13)	C15—C14—C19	118.64 (17)
C5—C6—O7	113.33 (15)	C14—C15—C16	120.39 (18)
C3—C6—H61	118.1	C14—C15—H151	119.4
C5—C6—H61	117.3	C16—C15—H151	120.3
O7—C6—H61	112.3	C15—C16—C17	120.55 (17)
C6—O7—C8	109.39 (14)	C15—C16—H161	120.0
C2—C8—O7	107.62 (14)	C17—C16—H161	119.4
C2—C8—O9	105.91 (13)	C16—C17—C18	119.59 (18)
O7—C8—O9	111.33 (16)	C16—C17—H171	119.4
C2—C8—H81	113.1	C18—C17—H171	121.0
O7—C8—H81	108.9	C17—C18—C19	119.92 (19)
O9—C8—H81	110.0	C17—C18—H181	120.5
C8—O9—C10	108.58 (13)	C19—C18—H181	119.6
O9—C10—O1	104.85 (14)	C14—C19—C18	120.89 (17)
O9—C10—C11	111.18 (15)	C14—C19—H191	119.8
O1—C10—C11	111.77 (14)	C18—C19—H191	119.3

### Hydrogen-bond geometry (Å, º)

**Table:**

*D*—H···*A*	*D*—H	H···*A*	*D*···*A*	*D*—H···*A*
C11—H111···N4	0.97	2.58	3.266 (3)	128
